# The Defensin NldefB as a Potential Target for Brown Planthopper Control Based on the Combination of RNA Interference and Fungal Insect Pathogen

**DOI:** 10.3390/insects16101041

**Published:** 2025-10-10

**Authors:** Chen-Ping Lan, Zhi-Guo Hu, Xiao-Ping Yu, Zheng-Liang Wang

**Affiliations:** 1 Zhejiang Provincial Key Laboratory of Biometrology and Inspection and Quarantine, College of Life Sciences, China Jiliang University, Hangzhou 310018, China; chenping_l808@163.com (C.-P.L.); 19511382056@163.com (Z.-G.H.); yuxiaoping19630306@163.com (X.-P.Y.); 2 Key Laboratory of Microbiological Metrology, Measurement and Bio-Product Quality Security (State Administration for Market Regulation), China Jiliang University, Hangzhou 310018, China

**Keywords:** defensins, *Nilaparvata lugens*, RNAi, *Metarhizium anisopliae*, immune regulation

## Abstract

Defensins are a class of small cysteine-rich cationic antimicrobial peptides (AMPs) that play vital roles in immune-regulating insect–microbe interaction, offering great potential for developing pest control approaches using RNA interference (RNAi) and insect pathogens. However, the biocontrol potential of defensins from the destructive rice pest *Nilaparvata lugens* (brown planthopper, BPH) remains largely unexplored. Here, we identified and functionally characterized a defensin-encoding gene *NldefB* in BPH. The open reading frame (ORF) of *NldefB* is 315 bp in length, encoding 104 amino acids with a conserved Knot1 domain. The qRT-PCR results showed that the transcription level of *NldefB* went upward with the increasing developmental stages, with the highest expressions in the female adults and their fat body. The expression of *NldefB* was continuously induced by bacterial pathogens but exhibited a pattern of initial increase followed by a decrease when challenged by a fungal pathogen *Metarhizium anisopliae*. RNAi-mediated silencing of *NldefB* significantly decreased the host survival rate, egg production and hatchability, as well as the capability to resist fungal infection. Additionally, *NldefB* suppression resulted in a significant increase in microbial loads. Our findings underscored that *NldefB* plays essential roles in regulating host development, pathogen defense, and microbial maintenance, providing a potential target for RNAi- and microbe-mediated BPH biocontrol.

## Simple Summary

The use of pathogenic and symbiotic microbes for pest biocontrol is a promising strategy for achieving sustainable and eco-friendly pest management. However, the robust immune defense system of insects limits the insecticidal efficacy of microbe-based biocontrol. Insect antimicrobial peptides (AMPs) represent a crucial family of potent immune effectors involved in host immune–microbe interaction, yet their potential for pest biocontrol remains largely unexplored. In this study, the gene *NldefB* which encodes an antimicrobial peptide defensin was identified and functionally characterized in *Nilaparvata lugens* (brown planthopper, BPH)*,* the most destructive rice pest. RNAi-mediated silencing of *NldefB* resulted in a marked reduction in survival rate, oviposition amount and hatchability, caused a significant enhancement in susceptibility to fungal infection, and led to a substantial increase in microbial load in BPH. Our findings underscored the critical roles of NldefB in mediating host physiology including reproductive development, pathogen defense, and microbial maintenance, highlighting that *NldefB* could serve as a target for BPH control by integrating RNAi and pathogenic/symbiotic microbes.

## 1. Introduction

The brown planthopper (BPH), *Nilaparvata lugens* Stål is a monophagous hemipteran insect reliant solely on rice for survival and is recognized as the most detrimental rice pest [1]. Biological control represents the optimal strategy for BPH management, with pathogenic and symbiotic microorganisms being the two important microbial resources in this strategy [2,3]. However, the insecticidal efficacy of microbe-mediated pest control is intricately linked to the innate immune defense responses of the host insects [4]. Therefore, comprehensive insights into the immune defense mechanism of BPH hold significant importance for the development of novel microbial control strategies against this rice pest.

Insect antimicrobial peptides (AMPs) play a crucial role in the innate immune system of insects and are primarily synthesized via the IMD and Toll signaling pathways [5,6]. To date, 397 insect AMPs have been determined according to the Antimicrobial Peptide Database (http://aps.unmc.edu/AP/, accessed on 20 August 2025). Insect AMPs are classified into different families based on their structural characteristics, including ceropins, defensins, attacins, drosocins, and diptericins [7,8]. Of those, defensins are a class of small cysteine-rich cationic proteins widely identified in diverse insect groups. Due to their broad-spectrum antimicrobial activities, they comprise a potent arm of the innate immune system in insects [9,10].

Insect defensins were firstly discovered in the flesh fly *Sarcophaga peregrina* [11], and subsequently identified in other insect species from Coleoptera, Hymenoptera, and Hemiptera [12,13,14,15]. Structural analysis reveals that they exhibit a relatively conserved structural skeleton, typically comprising 34–43 amino acid residues and 3–4 pairs of intramolecular disulfide bonds, which confer a cysteine-stabilized Cαββ motif [16]. Defensins are primarily known as effector molecules in innate immunity, helping the insect host resist pathogen invasion [17,18,19]. For instance, defensins from the honey bee *Apis mellifera* were found to exert potent antimicrobial activities against both bacterial and fungal pathogens (*Escherichia coli*, *Bacillus subtilis* and *Ascosphaera apis*), as well as parasites (*Nosema ceranae*) [19]. The classically considered mode of action of defensins is to disrupt cell membrane integrity and interfere with metabolic processes [20]. Recent studies demonstrated that defensins play a crucial role in the regulation of symbiont communities in insects [21,22,23,24]. Compared with the wild-type colony of the fruit fly *Drosophila melanogaster*, significant changes in the load and diversity of intestinal microbiota were detected in the defensin-deleted mutants [24].

Generally, sap-sucking hemipterans, like pea aphid and whitefly, appear to have a limited innate immune system [25,26,27]. For instance, the genome of the pea aphid *Acyrthosiphon pisum* is missing some immune effectors including defensins [27]. Based on the sequenced whole genome, BPH possesses a comprehensive immune defense system [15], yet limited research exists on its immune-related genes involving in pathogen defense and symbiont maintenance. In this study, we cloned and identified a defensin-encoding gene *NldefB* in BPH. The biological functions of *NldefB* in resisting fungal infection and maintaining symbiont homeostasis were investigated using RNA interference (RNAi). Our results provided a foundation for understanding the immune interaction mechanism between BPH and pathogenic/symbiotic microbes and will contribute to the development of microbe-mediated BPH control strategies.

## 2. Materials and Methods

### 2.1. Insects and Microbial Strains

The BPH population was maintained on rice seedlings (variety Xiushui 314) under controlled environmental conditions: 24 ± 1 °C, 70% ± 5% relative humidity, and a 14 h light/10 h dark photoperiod. *Escherichia coli* strain ATCC35150 and *Staphylococcus aureus* strain CMCC26003 were incubated in Luria–Bertani (LB) medium at 37 °C. *Metarhizium anisopliae* strain ARSEF456 (designated Ma456 hereafter) was cultured on Potato dextrose agar (PDA) plates at 25 °C.

### 2.2. RNA Extraction and cDNA Synthesis

Fifteen BPH adults were randomly collected and total RNA was extracted using the MiniBEST Universal RNA Extraction Kit (Takara, Dalian, China). RNA concentration and integrity were evaluated by a NanoDrop ND-200 microspectrophotometer (Thermo Scientific, Waltham, MA, USA) and 1% agarose gel electrophoresis, respectively. The cDNA was synthesized using the PrimeScript™ RT reagent Kit with gDNA Eraser (Takara, Dalian, China) and stored at −80 °C for use.

### 2.3. Gene Cloning and Structural Characterization of NldefB

The full-length of open reading frame (ORF) of *NldefB* was obtained by PCR using the cDNA template and specific primers (Table S1). PCR reactions were performed in a 50 μL mixture comprising 25 μL of 2 × Premix Taq, 1.5 μL each of sense and antisense primers, 2 μL of cDNA template and 20 μL of ddH_2_O. The purified PCR products were ligated into a T/A cloning vector pMD19-T (TaKaRa, Dalian, China) and transformed into competent cells of *E. coli* DH5α. Positive colonies were screened and sequenced by Zhejiang Youkang Biotechnology Co., Ltd, Hangzhou, China.

The physiochemical properties, structural domains, signal peptide region, and subcellular location of NldefB protein were analyzed using various bioinformatics tools as previously described [28]. Homologous sequences were searched by BLASTP (https://blast.ncbi.nlm.nih.gov/Blast.cgi) (accessed on 15 August 2025) and multi-aligned with ClustalW algorithm in MEGA X software. Phylogenetic analysis was performed using the maximum likelihood (ML) under the LG+G+I model that was inferred as the best model. Bootstrap values based on 1000 replications are indicated as percentages on the nodes [29].

### 2.4. Gene Expression Pattern Analysis of NldefB

The spatiotemporal expression pattern of *NldefB* was determined by collecting samples from various developmental stages (eggs, first–fifth instar nymphs and adults) and different tissues (fat body, gut, hemolymph, and ovary) as previously described [28]. Microbe-induced expression patterns were assessed by injecting 10 nL suspension of *E. coli*, *S. aureus* and *M. anisopliae* at a concentration of 1 × 10^7^ cells (conidia)/mL in 1 × PBS buffer (pH 7.0) into the fifth-instar BPH nymphs (*n* = 20) using a microinjector. Control nymphs were injected with an equal volume of 1 × PBS. Live BPH individuals were collected at 0, 12, 24, 36, 48, 60 and 72 h post-injection in triplicate.

Total RNA was extracted and the cDNA was synthesized following the above-described method. The quantitative reverse transcription PCR (qRT-PCR) was performed using the SYBR^®^ Premix Ex Taq^TM^ II kit (TaKaRa, Dalian, China) with the 18S rRNA gene of BPH as an internal reference. The reaction mixture contains 10 μL of 2 × SYBR^®^ Premix Ex Taq^TM^ II, 1 μL of each primer (Table S1), 2 μL of cDNA and 6 μL of ddH_2_O in a total volume of 20 μL. The reaction was conducted at 95 °C for 30 s, followed by 40 cycles of 95 °C for 5 s and 60 °C for 30 s. Relative expression of *NldefB* was analyzed using the 2^−∆∆Ct^ method [30].

### 2.5. Synthesis and Injection of Double-Stranded RNA (dsRNA)

The MEGAscript^®^ RNAi Kit (Ambion, Austin, TX, USA) was used to synthesize the dsRNA of *NldefB* (dsNldefB) with specific primers (Table S1). The dsRNA targeting the green fluorescent protein gene (dsGFP) served as a negative control. The quality and concentration of the synthesized dsRNA were assessed by agarose gel electrophoresis and a NanoDrop 2000 (Thermo Scientific, Waltham, MA, USA). The quantified dsRNAs were then diluted to a concentration of 2500 ng/μL with ddH_2_O and preserved at −80 °C.

The newly emerged BPH adults within 24 h were cryoanesthetized on ice before dsRNA microinjection. A volume of 25 nL of dsNldefB or dsGFP was injected into individual adults via thoracal segment using a microinjector (Eppendorf, Hamburg, Germany). The qRT-PCR was performed to check the RNAi efficacy at one, two and three days post-injection. Each treatment was replicated three times, with 20 adults per replicate.

### 2.6. Analysis of Survival and Fecundity After NldefB Silencing

Newly emerged adults were microinjected with dsNldefB or dsGFP as described above and then immediately transferred to fresh rice seedlings for feeding. The number of dead adults was recorded daily for ten consecutive days. Each treatment consisted of three replicates, with 50 adults per replicate. The survival rates were calculated and the survival curves were plotted by the Kaplan–Meier method and the log-rank test was used for comparison.

To evaluate the impact of *NldefB* knockdown on BPH fecundity, newly emerged unmated female adults were microinjected with dsNldefB. Each female was then paired with a healthy male in a long tube containing fresh rice seedlings. The rice seedlings were regularly replaced to meet the nutritional needs of BPH for feeding and egg-laying. The number of hatched nymphs from rice seedlings (including replaced rice seedlings) was monitored daily until no more nymphs hatched. Subsequently, the rice seedlings were dissected under a stereomicroscope to record the unhatched eggs. The total number of hatched and unhatched eggs was calculated and then the egg hatch rate was determined. Female adults injected with dsGFP served as the control group. Each pair of adults was considered as 1 replicate, with 30 replicates per treatment.

### 2.7. Analysis of Antifungal Defense After NldefB Silencing

To assess the effect of *NldefB* knockdown on host resistance to fungal infection, newly emerged adults were microinjected with dsNldefB or dsGFP and then immediately sprayed with 1 mL of Ma456 conidial suspension (1 × 10^8^ conidia/mL). The dsRNA-injected adults sprayed with an equal volume of 0.02% Tween 80 (used for suspending fungal conidia) were used as the negative control. The co-treated adults were transferred to fresh rice seedlings for feeding. Mortality was monitored daily for ten days, and the mortality rate was calculated. The values of median lethal time (LT_50_) for all bioassays were estimated using non-linear regression data analysis programs implemented in software GraphPad Prism 8. The statistical model of log (agonist) vs. normalized response (variable slope) was used for the parameter generation. Each treatment was replicated three times, with 50 adults per replicate.

To evaluate the effect of *NldefB* knockdown on antifungal activity of the host hemolymph, the hemolymph samples were collected from BPH adults co-treated with dsRNA injection and fungal infection for four days. Total DNA was extracted from the collected hemolymph using the DNeasy Tissue Kit (Qiagen, Hilden, Germany) according to the manufacturer’s instructions. The load of fungal hyphal bodies in the hemolymph was quantified by quantitative PCR (qPCR) using the SYBR^®^ Premix Ex Taq^TM^ II kit (TaKaRa, Dalian, China) with the M. anisopliae specific primers (Table S1). The 18S rDNA gene of BPH was used as an internal reference. The relative amount of fungal hyphal bodies in the hemolymph of adults co-treated with dsNldefB injection and Ma456 infection was calculated in comparison to the dsGFP+Ma456 co-treated control. Each treatment contained three replicates, with 100 adults per replicate.

### 2.8. Analysis of Symbiont Load After NldefB Silencing

To determine the effect of *NldefB* knockdown on the symbiont homeostasis maintenance, the newly emerged adults were injected with dsNldefB or dsGFP and then reared on fresh rice seedlings. At four days post-injection, live BPH individuals were collected and surface-sterilized with 75% ethanol followed by five rinses in ddH_2_O. Total DNA of each sample was extracted using the DNeasy Tissue Kit (Qiagen, Hilden, Germany). The abundance of bacteria and yeast-like symbiotes (YLS) were assessed by qPCR with universal bacterial 16S rRNA primers and YLS-specific primers (Table S1), respectively. The 18S rDNA of BPH was used as an internal reference. The relative abundance in the dsNldefB-injected adults was calculated in comparison to the dsGFP-injected control. Each treatment was conducted in triplicate, with 50 adults per replicate.

### 2.9. Statistical Analysis

DPS v7.05 software was utilized to perform statistical analysis of the experimental data [31]. Student’s *t*-test was used to evaluate significant differences between two groups, while multiple group comparisons were performed using one-way analysis of variance (ANOVA) followed by Tukey’s honest significant difference (HSD) post hoc test. The threshold for statistical significance was set at *p* < 0.05.

## 3. Results

### 3.1. Identification and Characterization of NldefB

**Figure 1:**
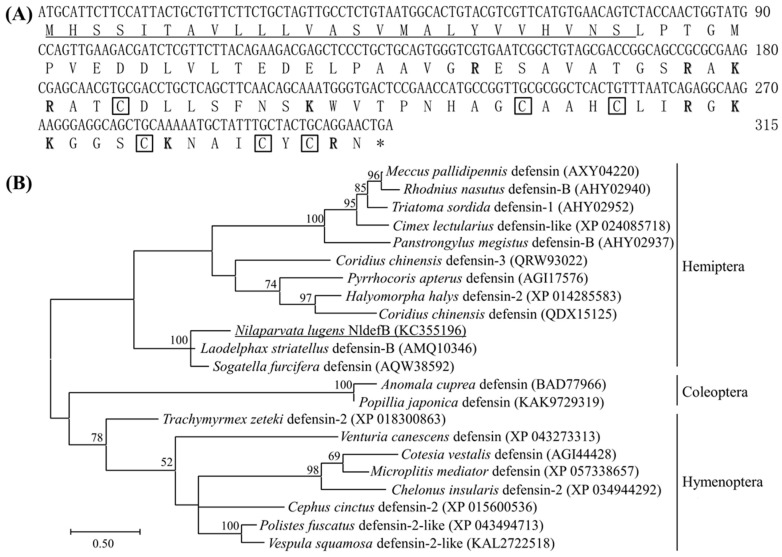
Structural and phylogenetic analysis of NldefB. (**A**) The cDNA and amino acid sequences of NldefB. Signal peptide sequence is underlined; The cationic amino acid residue is shown in bold and the conserved cysteine site is marked with a box; Asterisk indicates termination codon. (**B**) Phylogenetic tree of NldefB and defensins from other insects by maximum likelihood tree based on amino acid sequences. NldefB is underlined; The numbers at each branch point represents the percentage of the bootstrap values. Bootstrap values smaller than 50 are hidden. GenBank accession number for each insect defensin is parenthesized.

The full-length ORF of *NldefB* (GenBank accession number: KC355196) spans 315 bp and encodes a protein comprising 104 amino acids in length. The predicted molecular weight is 10.98 kD and the isoelectric point is 8.27. Sequence analysis revealed that NldefB contains ten positively charged residues (Lys^60/72/90/91/96^, Arg^49/58/61/88/103^). The N-terminal 1–25 amino acids serve as a signal peptide, with the cleavage site between Ser^25^ and Leu^26^. The prediction of subcellular localization showed that NldefB is a secreted extracellular protein. SMART analysis indicated a conserved Knot1 domain with six cysteine residues (Cys^64/81/85/95/100/102^) forming three intra-chain disulfide bonds present between residues 63 and 104 at the C-terminal (Figure 1A). A Blast search showed that NldefB shares the highest sequence homology (77.2%) with *Laodelphax striatelus* defensin-B (GenBank accession number: AMQ10346). The phylogenetic analysis showed that NldefB forms a monophylogenic group with its homologs in *L. striatellus* and *Sogatella furcifera* (Figure 1B).

### 3.2. Expression Patterns of NldefB

**Figure 2:**
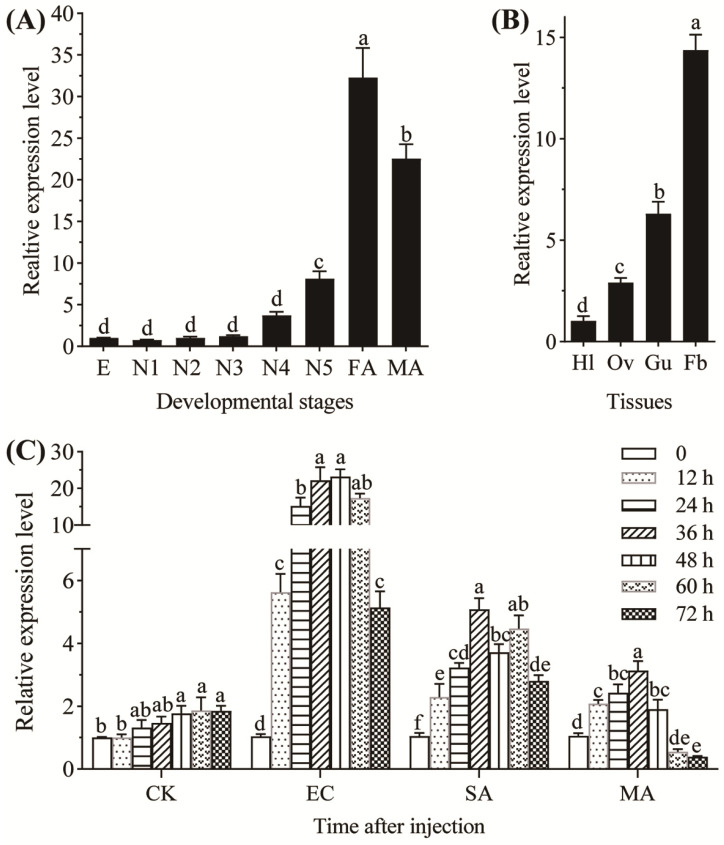
Analysis of expression pattern of *NldefB* in BPH. (**A**) Expression level of *NldefB* across different developmental stages of BPH. E: eggs (n = 100); N1-N5: first- to fifth-instar nymphs (n = 20); FA: female adults (n = 10); MA: male adults (n = 10). (**B**) Expression level of *NldefB* at different tissues of BPH. Hl: hemolymph; Ov: ovary; Gu: gut; Fb: fat body. A total of 100 newly emerged female adults were dissected as a replicate. (**C**) Expression level of *NldefB* at 0, 12, 24, 36, 48, 60 and 72 h after injection with different microbes in the fifth-instar nymphs of BPH (n = 100). CK: Control group; EC: *Escherichia coli*-treated group; SA: *Staphylococcus aureus*-treated group; MA: *Metarhizium anisopliae*-treated group. Each treatment contained three biological replicates. Different letters above bars indicate significant difference (*p* < 0.05, one-way ANOVA test).

The expression levels of *NldefB* gradually increased during BPH development. The transcripts in old nymphs (fourth- and fifth-instars) were significantly higher than those in the eggs and young nymphs (first-, second- and third-instars), and the highest expression level was observed in the female adults. Relative to the egg stage, the expressions in the female and male adults exhibit a 32.3-fold and 22.5-fold increase, respectively (Figure 2A). *NldefB* was expressed in all tested tissues of female adults, with the highest expression in the fat body, followed by the gut and ovary. The transcription level in the fat body is 14.1-fold higher than that in the hemolymph (Figure 2B). The expression of *NldefB* was significantly upregulated by both the Gram-negative *E. coli* and Gram-positive *S. aureus* within 72 h post injection. However, the induction effect by *E. coli* was more significant than that of *S. aureus*. For instance, the expression level of *NldefB* was markedly upregulated 14.6-fold after 24 h post injection with *E. coli*, while only a 3.1-fold increase was detected upon induction by *S. aureus*. The expression of *NldefB* induced by the fungal entomopathogen *M. anisopliae* displayed a pattern of initial increase followed by a decrease, with a peak occurring at 36 h post injection. Noticeably, the transcription level of *NldefB* was significantly suppressed by 62.9% after 72 h post-challenge (Figure 2C).

### 3.3. NldefB Silencing Decreased BPH Survival and Fecundity

**Figure 3:**
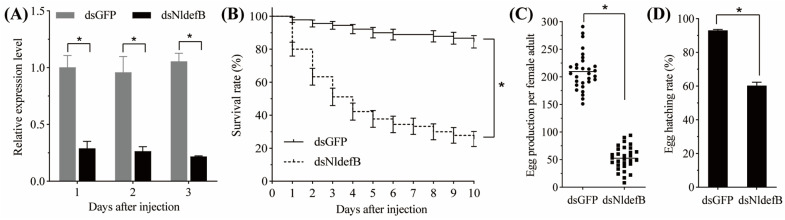
Effect of *NldefB* silencing on the BPH survival and reproduction. (**A**) Relative expression level of *NldefB* after dsNldefB injection. Each treatment had three biological replicates, and each replicate contained 20 newly emerged BPH adults. (**B**) The survival rates of newly emerged BPH adults during a ten-day period in the dsNldefB-injected group and the dsGFP-injected control group. Each treatment consisted of three replicates, with 50 adults per replicate. The survival analysis was performed using Kaplan–Meier plots (*p* < 0.05, log-rank chi-squared test). (**C**,**D**) The effect of *NldefB* silencing on the egg production and egg hatching rate. Each treatment contained 30 biological replicates, and each pair of adults was considered as one replicate. Asterisk (*) indicates significant difference determined between the dsNldefB-injected group and the dsGFP-injected control group (*p* < 0.05, *t*-test).

The RNAi efficacy in the newly emerged BPH adults via injection with dsNldefB is shown in Figure 3A. The qRT-PCR results revealed that the synthesized dsNldefB effectively suppressed the target gene expression. Compared to the control group injected with dsGFP, the transcription level of *NldefB* was markedly downregulated by 71.1%, 72.5% and 79.4% at one, two, and three days post-injection with dsNldefB, respectively. RNAi-mediated suppression of *NldefB* resulted in a significant reduction in survival rate of the newly emerged BPH adults. The survival rate of the dsNldefB-injected adults was 44.5% at seven days post-injection, which was significantly lower than that observed in the control group (88.9%) (Figure 3B). Additionally, the oviposition amount and hatchability were also significantly reduced following injection with dsNldefB (Figure 3C,D). Compared to the dsGFP-injected control, the egg production and egg hatching rate of the dsNldefB-injected group decreased by 74.8% and 60.3%, respectively.

### 3.4. NldefB Silencing Reduced BPH Resistance to Fungal Infection

**Figure 4:**
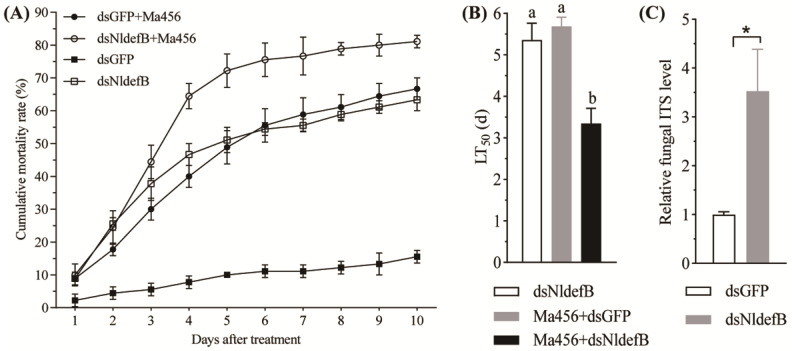
Effect of *NldefB* silencing on the BPH resistance to fungal infection. (**A**) Trends of cumulative mortality rates of BPH after combined treatment with *NldefB* silencing and *M. anisopliae* infection. The newly emerged adults were pre-injected with dsNldefB or dsGFP and then immediately sprayed with 1 mL of Ma456 conidial suspension (1 × 10^8^ conidia/mL), respectively. Each treatment consisted of three replicates, with 50 adults per replicate. (**B**) LT_50_ values of BPH after combined treatment with *NldefB* silencing and *M. anisopliae* infection. The values of LT_50_ for all bioassays were estimated using non-linear regression data analysis programs and the statistical model of log (agonist) vs. normalized response (variable slope) was used for the parameter generation. Different letters above bars indicate significant difference (*p* < 0.05, one-way ANOVA test). (**C**) The relative load of fungal hyphal bodies in the hemolymph of dsRNA-injected BPH nymphs at four days post-infection with *M. anisopliae*. The load of fungal hyphal bodies in the hemolymph was quantified by qPCR using the specific ITS primers of *M. anisopliae*. The 18S rDNA of BPH was used as an internal reference. The relative load in the hemolymph of adults co-treated with dsNldefB injection and Ma456 infection was calculated in comparison to the dsGFP+Ma456 co-treated control. Each treatment contained three biological replicates, and a total of 100 adults were dissected as a replicate. The asterisk (*) indicates significant difference (*p* < 0.05, *t*-test).

The cumulative mortality curves of the newly emerged BPH adults after the combination of dsRNA injection and fungal challenge are illustrated in Figure 4A. The host’s ability to resist *M. anisopliae* infection was significantly decreased by RNAi-mediated silencing of *NldefB*. For instance, the mortality rate was 75.6% in the dsNldefB-injected BPH at 6 days post-infection with *M. anisopliae*, while a significantly lower mortality of 55.6% was detected in the control BPH co-treated with dsGFP and fungal infection. As a result of the modeling analysis, The LT_50_ values for dsNldefB injection alone and co-treated with Ma456 infection were significantly different. The calculated LT_50_ value was 3.3 days for the dsNldefB+Ma456 co-treated group, which was 41.2% and 37.5% shorter than the estimates determined from dsNldefB injection alone (5.4 days) and dsGFP+Ma456 co-treatment (5.7 days), respectively (Figure 4B).

### 3.5. NldefB Silencing Boosted Fungal Proliferation in the Hemolymph of BPH

The fungal hyphal bodies in the hemolymph of the dsNldefB-injected BPH adults after topical infection with Ma456 were quantified via qPCR. The results showed a notable increase in the abundance of fungal hyphal bodies in the hemolymph of the dsNldefB-injected adults at four days post fungal infection in comparison with the control group. The fungal load in the hemocoel of the dsNldefB+Ma456 co-treated BPH was 3.5-fold increased relative to the control group (Figure 4C). These findings indicated that RNAi-mediated silencing of *NldefB* could boost fungal proliferation in the BPH hemolymph.

### 3.6. NldefB Silencing Enhanced Symbiont Load in BPH

**Figure 5:**
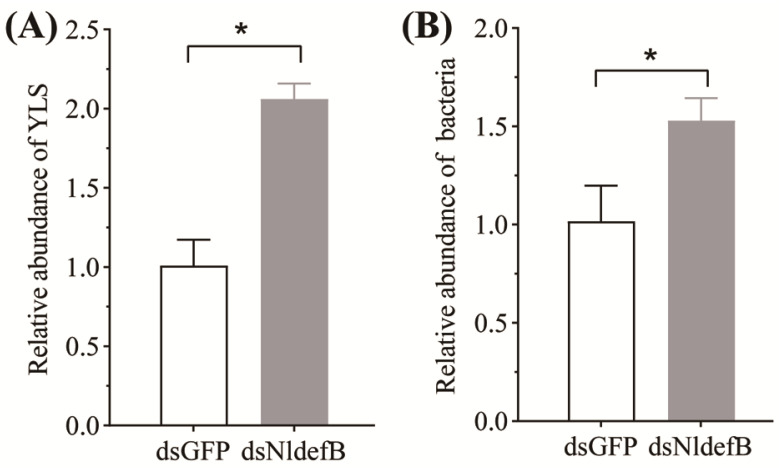
The effect of *NldefB* silencing on the load of yeast-like symbiotes (YLS) (**A**) and bacteria (**B**) in the body cavity of BPH. The asterisk (*) above bars indicates significant difference (*p* < 0.05, *t*-test). The abundance of YLS and bacteria were assessed by qPCR with YLS-specific primers and universal bacterial 16S rRNA primers, respectively. The 18S rDNA of BPH was used as an internal reference. The relative abundance in the dsNldefB-injected adults was calculated in comparison to the dsGFP-injected control. Each treatment consisted of three replicates, with 50 adults per replicate.

The qPCR data revealed a significant increase in YLS content within the body cavity of the dsNldefB-injected adults at four days post-injection. The abundance of YLS in this group was 2.4-fold higher than that in the dsGFP-injected control (Figure 5A). Similarly, the dsNldefB-injected BPH exhibited a significant increase in bacterial load in their bodies, as the total bacterial load was observed to be upregulated by 1.5 folds after *NldefB* gene silence (Figure 5B).

## 4. Discussion

The use of pathogenic and symbiotic microbes for pest biocontrol is a promising strategy for achieving sustainable and eco-friendly pest management [32,33]. However, the robust immune defense system of insects strongly limits the insecticidal efficacy of microbe-based biocontrol [4]. Insect AMPs represent a crucial family of potent immune effectors involved in host immune–microbe interaction, yet their potential for pest biocontrol remains largely unexplored. In this study, we identified and functionally characterized a defensin-encoding gene *NldefB*. Our findings revealed that NldefB is integral to host immune defense against fungal infection and host microbial homeostasis maintenance, as well as host survival and reproduction, highly suggesting its great potential as a target for BPH management by integrating RNAi and microbes.

Structurally, most insect AMPs are cationic basic polypeptides, comprising 2–8 positively charged amino acids such as arginine and lysine [34]. This cationic property allows these peptides to bind to the negatively charged microbial cell membranes, leading to membrane disruption and antimicrobial activity [20]. Sequence analysis revealed that NldefB contains five lysine and five arginine residues, affirming its cationic nature and strong affinity for microbial membranes. NldefB harbors six conserved cysteine residues, which can form three intramolecular disulfide bonds (C1–C4, C2–C5, C3–C6), characteristic of typical insect defensins. Structural predictions indicated that NldefB is a secreted protein containing a conserved Knot1 domain. Numerous studies have shown that proteins harboring this domain possess a wide range of biological activities, including antibacterial, antiviral, cytotoxic and insecticidal effects [35,36]. The high structural homology highlighted that NldefB is an evolutionarily and functionally conserved molecule with crucial roles in immune regulation.

The qRT-PCR analysis revealed that *NldefB* was constitutively expressed across all developmental stages of BPH. Expression levels were significantly higher in the older nymphs (fifth-instar) and adults compared to the eggs and young nymphs (first to fourth-instar), suggesting a crucial role for *NldefB* in the adult development. Similar expression patterns were observed in other insect species. For instance, the defensin-encoding gene *PxDef* in the diamondback moth *Plutella xylostella* was expressed at a low level at the young larval stage but markedly transcribed in the adult stage [18]. Tissue-specific expression profiling demonstrated that *NldefB* was highly expressed in the fat body and gut, two key immune responsive tissues in insects, manifesting that NldefB plays critical roles in regulating the immune responses of BPH. To date, the important contributions of defensins to insect antibacterial defense have been well documented [14,18,37]. Both Gram-negative (*E. coli*) and Gram-positive (*S. aureus*) bacteria could significantly induce the expression of *NldefB*, with *E. coli* exhibiting a more pronounced effect. These findings indicated that NldefB displays antibacterial activity mainly on Gram-negative bacteria and in a lesser extent on Gram-positive species. Such expression pattern was similar to the response of *BmdefensinB* in the silkworm *Bombyx mori* to microbial induction [37], but contrasted with *PxDef* in *P. xylostella*, which preferentially responded to Gram-positive bacteria [18]. Several studies have demonstrated that fungal entomopathogens also markedly upregulated the transcription of insect defensin genes [37,38]. For example, the expression of *BmdefensinB* in *B. mori* was continuously increased by fungal infection with *Beauveria bassiana* [37]. However, in contrast, *NldefB* expression was significantly suppressed by *M.anisopliae* infection within the extension of post-incubation time. A previous study demonstrated that *B. bassiana* infection in the mosquito *Anopheles stephensi* could significantly suppress defensin expression in the host gut, leading to gut flora imbalance and host mortality [39]. The mechanism underlying the downregulation of *NldefB* expression by *M. anisopliae* infection warrants further investigation.

RNAi-mediated silencing of *NldefB* resulted in a significant decrease in the survival rate, egg production and hatchability, indicating that NldefB is a key component in mediating host growth and reproduction of BPH. RNAi technology can effectively and specially suppress target gene expression, offering a great potential in pest control. This approach mainly relied on selecting key functional genes that are crucial for host survival and fecundity [40]. Our results indicated that NldefB could be a promising target for developing RNAi-based BPH control approaches. Fungal entomopathogens can infect and kill various agricultural hemipteran pests including aphids and whiteflies, via direct cuticle penetration [3,41]. Accumulative studies have demonstrated the compatible and synergistic effects of RNAi- and fungal entomopathogen-based biocontrol, as RNAi-mediated silencing of host immune-related genes could significantly augment the fungal virulence [42,43]. The significant increase in fungal load in the hemolymph of the *NldefB*-silenced adults after Ma456 challenge manifested that *NldefB* plays a vital role in host immune defense against fungal infection. Expectedly, the bioassays in the present study showed that *NldefB* suppression via RNAi markedly increased the insecticidal efficacy of Ma456 against BPH adults. This finding was in compliance with our recently published study which revealed that RNAi-mediated silencing of the Toll-like receptor (TLR) gene *NlToll1*, a key component of the Toll pathway involved in AMP production in BPH, significantly increased host susceptibility to fungal infection [28]. Therefore, our results highlighted that *NldefB* represents a promising shared target for integrated BPH management strategies by combining RNAi and fungal entomopathogens. The use of RNAi-mediated pest control necessitates a stable delivery system. Microbial agents, especially the fungal entomopathogens, are ideal vectors used for dsRNA delivery due to the synergistic effects of RNAi and fungal infection [44]. Future work should focus on constructing recombinant Ma456 strains that have expressed specific dsRNA targeting *NldefB* and evaluating their efficacy and safety in controlling BPH.

Insects harbor a diverse community of microorganisms that participate in a wide range of biological processes, including host growth and reproduction, nutritional metabolism and environmental adaptation [45]. Like other hemipteran pests such as aphids and whitefly, BPH contains an enormous number of symbiotic microbes [46]. YLS are dominant obligatory symbionts that primarily reside in the abdominal fat body of BPH and are transmitted vertically between host generations [47]. Extensive research has established their crucial role in host growth, development and reproduction. YLS can provide essential amino acids, sterols, and vitamins for BPH survival [48,49]. Additionally, diverse and rich bacterial communities, including *Arsenophonus* and *Wolbachia*, are detected in BPH, which participate in the regulation of host physiology including reproductive development, virulence variation, and insecticide resistance [50,51]. Disrupting microbial homeostasis inevitably caused the developmental and behavioral defects and finally resulted in the suppression of insect populations, conferring symbionts as a valuable microbial resource for pest biocontrol [33]. The insect’s immune defense responses, including the production of AMPs, play a crucial role in maintaining microbial balance [22,24]. Our results revealed a significant increase in the abundance of YLS and bacterial symbionts in the *NldefB*-silenced BPH, indicating that NldefB is an important regulator in maintaining microbial hemostasis in this rice pest. This microbial imbalance may be a key factor contributing to the substantial decline in survival and fecundity observed in the *NldefB*-silenced BPH.

Collectively, the *NldefB* gene encodes a typical antimicrobial peptide belonging to the defensin family that is essential for host immune modulation. RNAi-mediated knockdown of *NldefB* resulted in a marked reduction in survival rate, oviposition amount, and hatchability. Furthermore, *NldefB* silencing significantly decreased the resistance to fungal infection, as well as highly increased the microbial load in the body cavity of BPH. Our findings underscored the critical roles of NldefB in mediating host physiology including reproductive development, pathogen defense and microbial maintenance, highlighting that *NldefB* could serve as a target for BPH control by integrating RNAi and pathogenic/symbiotic microbes.

## Data Availability

The data presented in this study are available in the article and Supplementary Materials. Further inquiries can be directed at the corresponding authors.

## Supplementary Materials

The following supporting information can be downloaded at: https://www.mdpi.com/article/10.3390/insects16101041/s1, Table S1. Specific primer pairs used in this study.
