# Supplementary material for: The Defensin NldefB as a Potential Target for Brown Planthopper Control Based on the Combination of RNA Interference and Fungal Insect Pathogen
Source: Insects. 2025 Oct 10;16(10):1041. doi: 10.3390/insects16101041 (PMC12564868; doi:10.3390/insects16101041)
Supplement: Supplementary file 1 [file insects-16-01041-s001.zip › insects-3879792-supplementary.pdf]

Table S1. Specific primer pairs used in this study.

Primers	Sequences (5′-3′)	T_m value (°C)	Product length	Purpose
cNldefB-F	ATGCATTCTTCCATTACTGCTGTTC	61.9	315 bp	Gene cloning
cNldefB-R	TCAGTTCCTGCAGTAGCAAATAGCA	63.5		
qNldefB-F	TGCCTCTGTAATGGCACTGTA	56.7	192 bp	Gene expression analysis
qNldefB-R	GGAGTCACCCATTTGCTGTTG	60.2		
q18S-F	GTAACCCGCTGAACCTCC	54.8	170 bp	
q18S-R	GTCCGAAGACCTCACTAAATCA	61.7		
dsNldefB-F	GGATCCTAATACGACTCACTATAGGCTTCCATTACTGCTGTTCTTCTGCT	61.6	306 bp	dsRNA synthesis
dsNldefB-R	GGATCCTAATACGACTCACTATAGGAGTTCCTGCAGTAGCAAATAGCAT	61.3		
dsGFP-F	GGATCCTAATACGACTCACTATAGGCAGTGCTTCAGCCGCTACC	59.1	495 bp	
dsGFP-R	GGATCCTAATACGACTCACTATAGGCATGCCGAGAGTGATCCC	56.1		
qITS-F	GTAGCCCCTCAAGTCCCCTGC	64.7	141 bp	Quantitative microbial analysis
qITS-R	GTTGGCTCCTGTTGCGAGTGC	65.3		
qYLS-F	TCCCTCTGTGGAACCCCAT	59.4	164 bp	
qYLS-R	GGCGGTCCTAGAAACCAACA	60.0		
q16S-F	CGGCAACGAGCGCAACCC	67.7	146 bp	
q16S-R	CCATTGTAGCACGTGTGTAGCC	60.7		
